# Pb Stress and Ectomycorrhizas: Strong Protective Proteomic Responses in Poplar Roots Inoculated with *Paxillus involutus* Isolate and Characterized by Low Root Colonization Intensity

**DOI:** 10.3390/ijms22094300

**Published:** 2021-04-21

**Authors:** Agnieszka Szuba, Łukasz Marczak, Rafał Kozłowski

**Affiliations:** 1 Polish Academy of Sciences, Institute of Dendrology, Parkowa 5, 62-035 Kórnik, Poland; 2 Polish Academy of Sciences, Institute of Bioorganic Chemistry, Noskowskiego 12/14, 61-704 Poznań, Poland; Lukasz.Marczak@ibch.poznan.pl; 3 Institute of Geography and Environmental Sciences, The Jan Kochanowski University, 25-406 Kielce, Poland; rafalka@ujk.edu.pl

**Keywords:** citrate biosynthesis, dilution effect, ectomycorrhiza, heavy metals, proteomic and metabolomics

## Abstract

The commonly observed increased heavy metal tolerance of ectomycorrhized plants is usually linked with the protective role of the fungal hyphae covering colonized plant root tips. However, the molecular tolerance mechanisms in heavy metal stressed low-colonized ectormyocrrhizal plants characterized by an ectomycorrhiza-triggered increases in growth are unknown. Here, we examined *Populus × canescens* microcuttings inoculated with the *Paxillus involutus* isolate, which triggered an increase in poplar growth despite successful colonization of only 1.9% ± 0.8 of root tips. The analyzed plants, lacking a mantle—a protective fungal biofilter—were grown for 6 weeks in agar medium enriched with 0.75 mM Pb(NO_3_)_2_. In minimally colonized ‘bare’ roots, the proteome response to Pb was similar to that in noninoculated plants (e.g., higher abundances of PM- and V-type H^+^ ATPases and lower abundance of ribosomal proteins). However, the more intensive activation of molecular processes leading to Pb sequestration or redirection of the root metabolic flux into amino acid and Pb chelate (phenolics and citrate) biosynthesis coexisted with lower Pb uptake compared to that in controls. The molecular Pb response of inoculated roots was more intense and effective than that of noninoculated roots in poplars.

## 1. Introduction

Heavy metals, including lead (Pb), constitute one of the most dangerous environmental stressors affecting the whole plant physiology. Pb, either directly or via Pb-triggered oxidative stress, disrupts key plant cell macromolecules, resulting in DNA mutations, lipid peroxidation or improper protein folding and aggregation [1,2,3,4]. As a consequence, Pb exposure results in leaf chlorosis, decreases in root mass and the inhibition of whole plant growth [2,5,6,7,8,9].

Plants have developed numerous tolerance mechanisms aimed at decreasing the negative impact of free Pb^2+^ ions [10,11,12,13]. One of the most important stress-tolerance-increasing mechanisms is colonization by ectomycorrhizae (ECM), a class of obligate symbiotic fungi associated with numerous vascular plants [14,15,16,17,18,19].

The main advantage of this mutual association is increased water and nutrient transfer into the plant partner in exchange for the transfer of carbohydrates to the fungal partner [20,21,22]. It is commonly believed that this resource exchange occurs at the root tips, which are covered and partly penetrated (the epidermis and cortex, which are known as the Hartig net) during functional ectomycorrhizal symbiosis by the fungal hyphae to create a new special organ, the colonized root tip [17,18,21,23].

This additional layer created by the fungal mantle plays a very important role in the increased tolerance of ECM plants against heavy metals (HMs; [8,14,21,24,25,26,27,28,29]. ECM fungi release large amounts of various HM chelating agents, such as low-molecular-weight organic acids, phenolic compounds or low molecular weight proteins, all of which create a monolayer around the colonized root to generate the first biofilter that blocks Pb access into the plant cell (e.g., [10,25,28]). Next, heavy metals are detoxified in fungal cells; the processes of Pb binding to chitin in the fungal cell wall or sequestration in fungal vacuoles usually result in significantly decreased Pb in root plant cells [8,14,24,25,26,27,28,29]. Despite some exceptions (fungal exudates may decrease the pH in the rhizosphere and increase Pb availability; [10,29,30]), it is commonly believed that ECM fungi generate an efficient Pb biofilter [8,11,14,18,24,25,28,31].

However, ectomycorrhizal strains significantly differ in root colonization ratios [18,28], and the protective effect of ECM in Pb-exposed plants may also be a direct consequence of the increased plant growth triggered by the symbiotic fungi. Usually, improved nutrition and water access results in improved CO_2_ assimilation and antioxidative and carbohydrate status, which directly increases host resistance to impending HM stress [8,28,29,32,33,34,35]. Moreover, symbiotic partners influence the molecular activity of plant hosts by modifying the activity of genes involved in defense, energy metabolism, and cell wall biosynthesis, which altogether increases HM tolerance of ectomycorrhizal (M) plants [8,29,35]. These factors may increase HM tolerance regardless of root colonization ratio, but there is no molecular data available on the heavy metal stress response of plants devoid of a protective fungal biofilter (mantles).

All these improvements also, in most cases, increase the biomass of mycorrhized plants, which generates an additional effect: HM particles are dispersed within the increased root cell volume of M plants. It has been reported that high HM influx may be less harmful in bigger M plants [29,36]. Because ectomycorrhizal fungi may promote plant host growth independently of the degree of root colonization ([37,38], Szuba, unpublished data), ECM-triggered increases in plant mass volume may play an important role in the increased HM tolerance of M plants [36].

Nevertheless, the molecular adjustments in Pb-treated plants triggered by inoculation with ectomycorrhizal fungi remain largely unknown. Our goal was to highlight these unknown mechanisms, especially the very poorly recognized role of the effect of heavy metal dilution on the increased plant mass volume of M plants.

In most cases, M roots are covered by the fungal mantle and the protective effects of ECM during HM stress were analyzed previously almost exclusively in plants colonized by ECM fungi, which therefore had a protective fungal biofilter [14,15,16,17,18,19]. Furthermore, ECM usually results in increases in plant host biomass; thus, distinguishing between the protective effects of ECM caused by the fungal biofilter and the exclusive effects of the increased growth in inoculated, HM-stressed plants is a challenging task.

We hypothesized that, in M plants colonized with a low colonization ratio mycorrhiza, roots would be directly exposed to Pb but simultaneously affected at the molecular level by the presence of the fungal partner (in the form of the external mycelium). Consequently, in such ‘bare’ or minimally colonized M roots, the Pb response will be significantly different than that in non-inoculated (NM) plants. The interesting and open question is whether the molecular response to Pb exposure will be enhanced e.g., due to intensified Pb detoxification/exclusion triggered by the presence of the fungal partner.

In our study, we analyzed *Populus × canescens* inoculated with a *Paxillus involutus* isolate, which significantly improved plant host growth regardless of the low root colonization ratio (Szuba, unpublished data). Such a combination provides a unique opportunity to analyze the molecular responses of ectomycorrhizal plants to HM stress influenced by the ECM-triggered increase in biomass but not by the presence of biofilters created by fungal hyphae.

In the present experiment, a high-throughput gel-free proteomic tool (combined with a metabolomic approach) was used to examine the unknown molecular responses of poplars minimally colonized with ectomycorrhizal fungi to Pb exposure.

## 2. Results

### 2.1. Poplar Biometrics and Root Colonization Ratio

**Figure 1:**
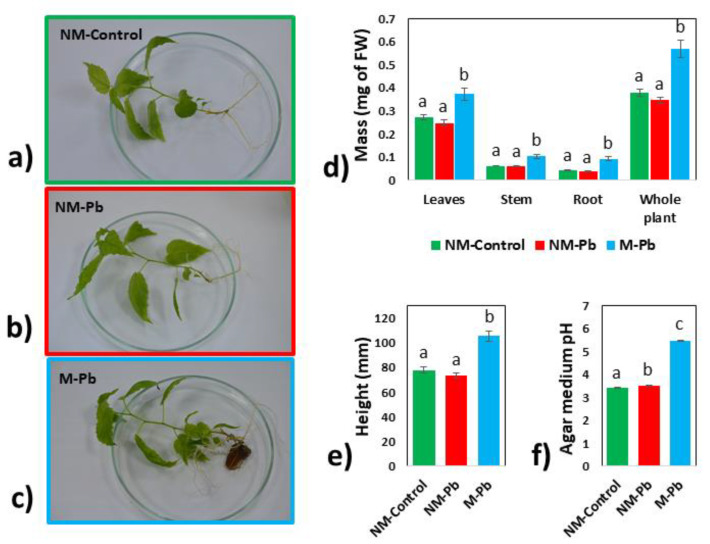
Biometrics and growth medium acidity. Representative images of NM-Control (**a**), NM-Pb (**b**) and M-Pb (**c**) poplar microcuttings. Fresh masses of leaves, stems, roots and whole plant mass presented for all treatments (**d**); (n = 16). Height of analyzed poplars (**e**); (n = 16). Acidity of the agar growth medium collected after six weeks of poplar growth (**f**); (n = 4). The mean values ± SE are presented. Different letters represent significant differences according to the HSD post hoc test.

After six weeks of plant growth (Figure 1a–c), Pb-exposed plants inoculated with *Paxillus involutus* (M-Pb) were characterized by larger mass (Figure 1d) and height (Figure 1e) compared to NM-Control and NM-Pb poplars (Figure 1a–e).

The acidity of the agar growth medium significantly differed between all treatments and was lower for the Pb treatments, with a dramatic decrease for the M-Pb treatment (Figure 1f).

**Figure 2:**
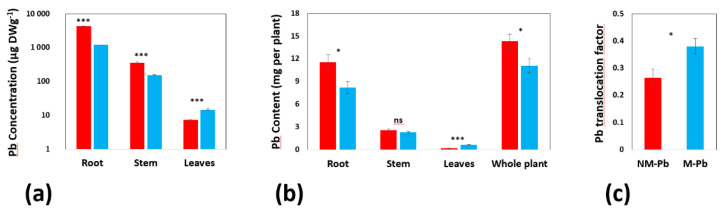
Pb concentrations (**a**); (n = 6) and contents (**b**); (n = 16) calculated for roots, stems and leaves from the NM-Pb and M-Pb treatment groups. The Pb translocation factor (**c**); (n = 16). The mean values ± SE are presented. Asterisks (ns = not significant; * *p* < 0.05; *** *p* < 0.001) represent significant differences according to the T test.

The highest Pb levels were found in roots compared to those in aboveground poplar tissues independently of root colonization (Figure 2a,b). In addition, Pb concentrations (μg DWg^−1^; Figure 2a) and contents (mg per plant; Figure 2b) in roots and stems (stem content was insignificant) were lower in M-Pb poplars than in NM-Pb poplars. In contrast, in leaves of M-Pb poplars, Pb was found at higher concentrations and with higher contents compared to that in leaves of NM-Pb poplars (Figure 2a,b). This increase resulted in a higher translocation factor in M-Pb poplars (compared to that in NM-Pb poplars; Figure 2c) but had no impact on the whole poplar Pb content, which was lower in M-Pb poplars (Figure 2b).

*Paxillus involutus* grew well on the agar surface (Figure S1), but the root colonization ratio was very low. The mycorrhized root tips were about 11% of the total (1.9% ± 0.8 of root tips were fully colonized, and 9.6% ± 2.4 of root tips were covered by fungi but lacked a fully developed Hartig net (changed root tips; [18]), and the phenotype of most M-Pb roots did not differ from that of NM roots, except that the M-Pb roots were larger (Figure 1a–c, Figure S1). The relative abundance of ergosterol (indicator of the fungal biomass not detected in stems and leaves nor in the NM roots; data not shown) in the M-Pb treatment group, which was estimated on the basis of comparison with *P. involutus* hyphae (established as 100%), was 0.8% ± 0.8 of the root biomass. The root samples consisted of more than 99% plant tissue.

### 2.2. Root Proteome

First, the root protein abundances detected in NM-Pb poplars were compared to those in the NM-Control treatment group to evaluate the ‘basic’ effect of Pb on the root proteome. Our main goal was to highlight the molecular differences between noninoculated and inoculated plants exposed to Pb^2+^ ions. For this reason, we performed proteome comparisons of M-Pb plants with two plant groups: NM-Controls and NM-Pb poplars.

#### 2.2.1. NM-Pb vs. NM-Control

**Figure 3:**
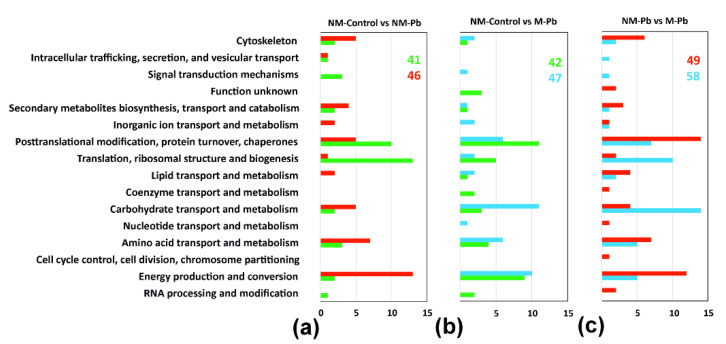
Protein classification according to COG (Clusters of Orthologous Groups) classes. Differentially abundant proteins detected in three independent two-sample T tests, NM-Control vs. NM-Pb (**a**), NM-Control vs. M-Pb (**b**) and NM-Pb vs. M-Pb (**c**), were divided into the COG classes according to the proteins that were more abundant in a particular treatment group. The following color codes were used for all classes: green columns, more abundant in NM-Control roots; red columns, more abundant in M-Pb roots; blue column, more abundant in M-Pb roots. The total number of upregulated proteins in a given treatment group and COG class (x-axis) are given.

In noninoculated poplars grown for 6 weeks in agar medium enriched with 0.75 mM Pb(NO_3_)_2,_ 87 proteins were differentially abundant (compared to NM-Controls; Table S1). In NM-Pb plants, we found a few proteins associated with stress responses, including an increased abundance of alcohol dehydrogenases (Figure 3a and Table S1; [39]). More proteins involved in carbohydrate metabolism were also found in the NM-Pb than in the NM-Control plants (Table S1). PM- and V-type ATPases, which are involved in the generation of the H^+^ gradient necessary for membrane transport of numerous compounds and ions, including Pb^2+^ (classified into the ‘energy production and conversion’ Clusters of Orthologous Groups (COG)), were repeatedly identified as being more abundant in NM-Pb roots (Figure 3a and Table S1), but 14-3-3 proteins were less abundant (Table S1). Enzymes involved in protein turnover, especially protein biosynthesis, as well as photosynthetic proteins (Table S1), were less abundant in NM roots exposed to Pb (Figure 3a and Table S1). Actin was less abundant, whereas tubulin was more abundant in NM-Pb roots than in NM-Controls (Figure 3a and Table S1).

#### 2.2.2. M-Pb vs. NM-Control: Pb Molecular Sensitivity in M-Pb Roots

The number of differentially abundant proteins between NM-Controls and M-Pb plants was only slightly higher than that observed during the NM-Control vs. NM-Pb comparison (89; see Figure 3b and Table S1), and numerous COG were affected similarly in both Pb treatment groups compared to NM-Control plants. For example, proteins involved in ‘carbohydrate transport and metabolism (including glycolytic enzymes; Table S1)’ or ‘energy production and conversion’ were more abundant (Figure 3a vs. Figure 3b), whereas photosynthetic proteins were less abundant in Pb-treated plants independent of *P. involutus* inoculation (compared to Controls; Table S1). Similar to NM-Pb, HSP80 and HSP90 were more abundant in M-Pb roots, but we also detected a higher abundance of proteins related to biotic stress (wound-induced protein and endochitinase; Table S1). HSP70s were more numerous in NM-Control roots (compared to both Pb treatment groups; Table S1). Proteins involved in protein turnover were less abundant in M-Pb poplars (similar to the NM-Pb to NM-Control comparison), but those changes were not as clear as in those in noninoculated plants (Figure 3); several proteins involved in protein biosynthesis and degradation were also more abundant in M-Pb plants (compared to those in NM-Controls; Table S1).

ATP synthases were found to be less abundant in M-Pb roots than in NM-Controls (Table S1). The influence of Pb on H^+^ ATPases was more intense in M poplars than in NM plants; all detected membrane pumps (both PM- and V-type H^+^ ATPases) were more abundant in M-Pb roots (Figure 3b; Table S1).

Some signals of increased lipid metabolism were also found in all Pb-treated plants (Figure 3), including a higher abundance of phospholipase D (Table S1). Aspartate aminotransferase (enzyme involved in glutamate/aspartate metabolism) was more abundant, whereas glutamine synthetase (which catalyzes glutamine/glutamate transformation) was less abundant in M-Pb roots (Table S1). Finally, proteomic signals of decreased lignin biosynthesis were found in M-Pb plants (Table S1).

#### 2.2.3. M-Pb vs. NM-Pb: Effect of Inoculation and Increased Biomass of M-Pb Plants

The comparison of the two Pb-exposed treatment groups revealed distinct differences in the root molecular status between NM- and M-Pb-stressed poplars due to the effects of fungal inoculation and the ECM-triggered biomass increase. Proteins that differed in abundance between M-Pb an NM-Pb treatments were more numerous (107), and the differences between the compared proteomes were different than those found when comparing the Control and Pb treatments (Figure 3c and Table S1). In M-Pb plants, signals of increased protein turnover, especially increases in ribosomal proteins, were found (Figure 3c, Table S1), but HSP70 chaperones were less abundant in inoculated poplars exposed to Pb (Table S1). Generally, stress-related proteins were more frequently detected as differentially abundant in this comparison (Table S1), but no clear trends were observed (e.g., wound-induced proteins were more abundant, but osmotic-like proteins were less abundant in M-Pb roots), except for redox-related proteins, which were less abundant in M-Pb roots (compared to the NM-Pb treatment group; Table S1). In M-Pb plants, PAL was less abundant as well as enzymes involved in lignin biosynthesis (Table S1). In M-Pb roots, glycolytic proteins were more abundant than in the NM-Pb treatment group, but enzymes involved in further energy production and conversion were less abundant in inoculated plants exposed to Pb (Figure 3c). In the M-Pb roots, aspartate aminotransferase was more abundant and glutamine synthetase was less abundant than in the NM-Pb roots, similar to that observed in the NM-Controls (Table S1). Finally, phospholipase D was more abundant and tubulin was less abundant in M-Pb plants (Figure 3c and Table S1).

#### 2.2.4. Proteins Detected as Variable in All Three Proteomic Comparisons 

**Figure 4:**
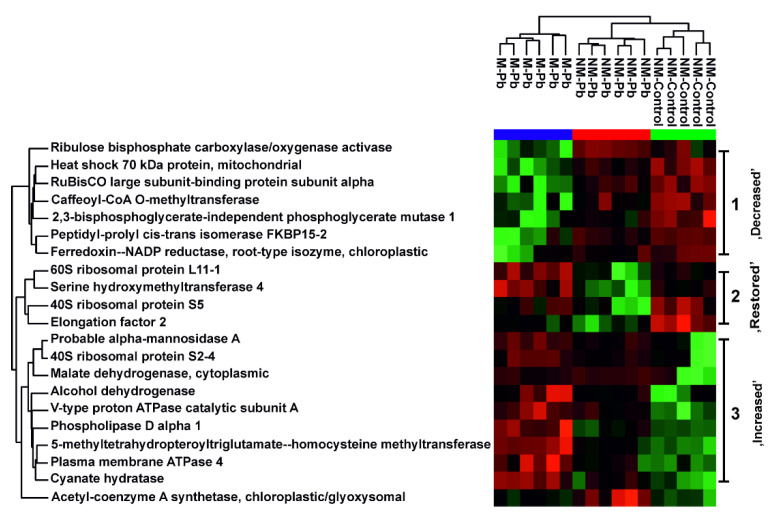
Common root proteins. The heat map analysis combined with hierarchical cluster analysis showing the ion intensities of proteins identified as differentially abundant according to all three two-sample test T tests: NM-Control vs. NM-Pb, NM-Control vs. M-Pb and NM-Pb vs. M-Pb. Green, minimal abundance; red, maximal abundance. Ion intensities were log2-transformed, batch-corrected and Z-scored for each row. Three protein clusters are clearly visible: proteins with decreased abundance in the series NM-Control→NM-Pb→M-Pb, proteins with similar abundance in NM-Control and M-Pb roots but with decreased abundant in NM-Pb poplars (so-called ‘restored’ proteins) and proteins with increased abundance in the series NM-Control→NM-Pb→M-Pb.

In the present study, 21 proteins were identified as differentially abundant in all three proteomic comparisons (Figure 4), which corresponds to 20–25% of the affected proteins. These varyingly abundant proteins could be divided into three subgroups based on their abundances in the NM-Control→NM-Pb→M-Pb series. Among the proteins characterized by decreased abundance in the presence of Pb, especially in M-Pb roots, we detected enzymes involved in photosynthesis, lignin biosynthesis and a previously identified 2,3-bisphosphoglycerate-independent phosphoglycerate mutase, which is a late glycolytic enzyme (Figure 4). The most numerous proteins were those with increased abundance in NM-Control→NM-Pb→M-Pb series. Among these proteins were enzymes involved in lipid metabolism, cytoplasmic malate dehydrogenase, alcohol dehydrogenase and PM- and V-type membrane ATPases (Figure 4).

Four proteins with significantly varying abundances in all three comparisons were decreased in NM-Pb roots, but, in M-Pb plants, their abundances were similar to those in NM-Controls (so-called ‘restored proteins’, Figure 4). These proteins included a serine hydroxymethyltransferase that catalyzes the interconversion of serine and glycine and proteins involved in ribosome functioning (Figure 4).

### 2.3. Metabolome Adjustments (GC MS/MS Study)

**Figure 5:**
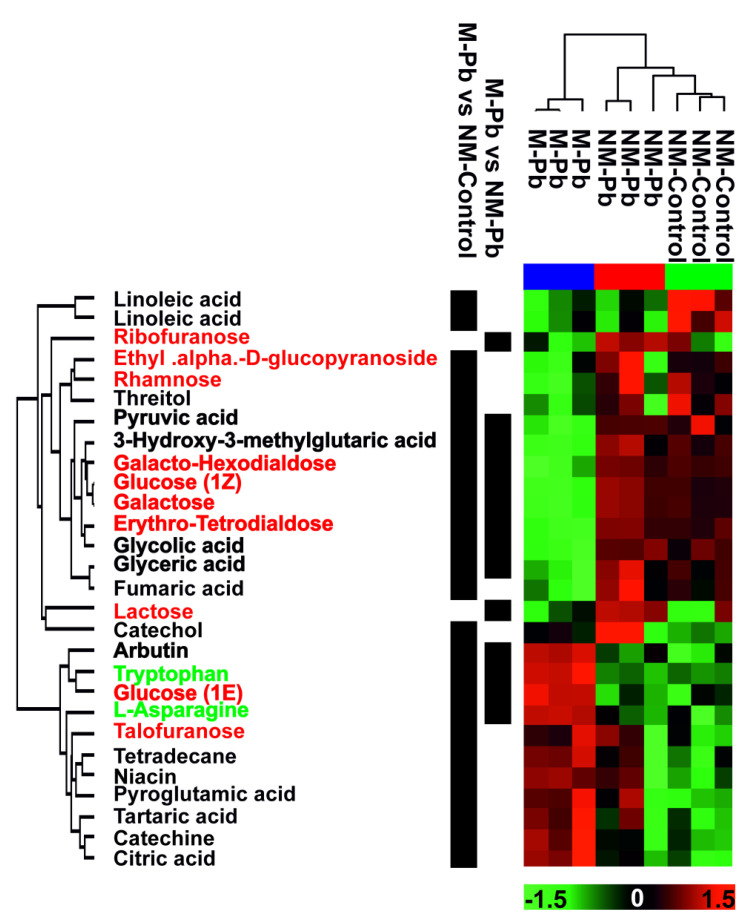
Metabolomic data. Heat map analysis combined with hierarchical cluster analysis showing poplar root ‘primary metabolites’ (GC MS/MS experiment) that are differentially abundant (*p* < 0.05) according to the multisample ANOVA test. Green, minimal abundance; red, maximal abundance. Ion intensities were log2-transformed, batch-corrected and Z-scored for each row. None of the differentially abundant compounds were detected between NM-Controls and NM-Pb roots (according to the T test). Compounds differently abundant according to the two remaining T tests (NM-Control vs. M-Pb and NM-Pb vs. M-Pb) are marked. Green font: amino acids, red font: carbohydrates. Bold: compounds identified as differently abundant according to both T tests (NM-Control vs. M-Pb and NM-Pb vs. M-Pb).

The nontargeted GC MS/MS study aimed to analyze amino acids, carbohydrates, organic acids and other primary metabolites, but other compounds, including lipidic or phenolic compounds, were also identified (Figure 5). Among the 280 detected compounds, there were no differently abundant compounds between the NM-Control vs. NM-Pb treatment groups, 73 (26 identified and 47 unknown compounds) differently abundant compounds between the NM-Control vs. M-Pb treatment groups and 39 (including 14 identified) between the NM-Pb vs. M-Pb groups (Table S2). According to the hierarchical analysis of compounds selected on the basis of the multisample ANOVA, the major factor influencing the analyzed metabolome was inoculation with *P. involutus* rather than exposure to Pb (data for the identified compounds are presented in Figure 5). The numerous differently abundant carbohydrates were less abundant in M-Pb roots compared to NM-Control roots (and also to NM-Pb roots, for the majority; Figure 5). In M-Pb plants, pyruvic acid, the end product of glycolysis, and glycolic and glyceric acid were less abundant (compared to both NM treatment groups). Citric acid was more abundant in Pb-treated roots (significant only in M-Pb roots; Figure 5) along with the massive upregulation of citrate synthase (Table S1). Flavonoids and amino acids were generally more abundant in M-Pb plants than in both NM treatment groups, especially when compared to the levels in NM-Controls (Figure 5).

## 3. Discussion

### 3.1. Molecular Response to Pb Exposure in NM and M Poplar Roots—Similarities

In roots inoculated with *P. involutus* characterized by a very low colonization ratio, most of the root surface was not covered with fungal hyphae and thus was deprived of fungal biofilters influencing the plant cell response to heavy metal stress [8,14,24,25,26,27,28,29]. For this reason, when the differences between the control and Pb-exposed poplar roots were analyzed, the molecular adjustments were, in many aspects, very similar in the noninoculated and inoculated plants (for the plant molecular adjustments caused by the analyzed *P. involutus* strain under control conditions see [40]).

In NM-Pb and M-Pb roots, increased abundances of PM- and V-type H^+^ ATPases were observed. H^+^ ATPases play key roles in heavy metal tolerance [12,19,41,42,43,44] because the proton gradient is necessary for the transport of organic compounds needed for Pb sequestration in the rhizosphere as well as for Pb plasma membrane and tonoplast transport and sequestration in vacuoles [3,44]. These mechanisms seem to be efficient in all Pb-exposed poplars since we did not observe numerous proteomic signals of stress responses, including HSP overexpression or anti-free-radical activity, which are some of the most frequently observed proteomic responses to heavy metal stress [6,45,46,47,48,49,50,51]. The relatively low mobilization of anti-free-radical enzymes suggests the presence of low free radical concentrations in Pb-exposed roots.

Interestingly, in both NM-Pb and M-Pb roots, decreased abundances of proteins involved in ribosome activity were observed, indicating decreased protein biosynthesis [6, 46]. Protein biosynthesis may be disrupted by strong Pb-induced stress [5]. However, in the present study, no significant negative phenotypic changes were observed due to Pb; no decreased growth, mass or leaf chlorosis was observed [7,52]. The poplar microcuttings were exposed to mild Pb-induced stress (most probably Pb^2+^ ions were precipitated in the MS medium rich in phosphates and sulfates; [18,29]). In the present experiment, decreased protein turnover was rather related to the low Pb dose, and most importantly to the chronic nature of Pb exposure. To reduce energy-consuming processes such as protein biosynthesis [3,47,53] only proteins necessary to provide metabolome stability are upregulated. Decreased or unchanged protein turnover was previously observed in plants exposed to prolonged stress [49,54,55]. Decreased protein biosynthesis may be indirectly confirmed by a decrease in HSP70 abundance. HSP chaperones are frequently upregulated under stress conditions [3,45,54] but are necessary mainly for protein maturation (also under physiological conditions; [45]). Such a decrease in HSP70 suggests a decreased number of biosynthesized and damaged proteins, which may be linked with low ROS levels in Pb-treated poplar roots, as suggested by our proteomic results.

In contrast, we detected more abundant HSP80 and HSP90. These chaperones are rather linked with stress adaptation and reducing negative effects of environmental perturbations than with the acute HM stress response [45,55,56]. The decrease in protein turnover was less obvious in M-Pb plants because in inoculated plants, the effect of the plant response to chronic Pb exposure coexisted with the continuous influence on the plant proteome (plant metabolism) of the presence of the symbiotic partner [57,58,59,60,61].

### 3.2. Paxillus involutus—What Difference Does Inoculation by ‘Low-Colonizer’ Make in the Plant Pb Response?

Despite many similarities, as hypothesized, the Pb response differed between noinoculated and inoculated poplars, involving a direct influence by fungal inoculation and endochitinase induction.

**Figure 6:**
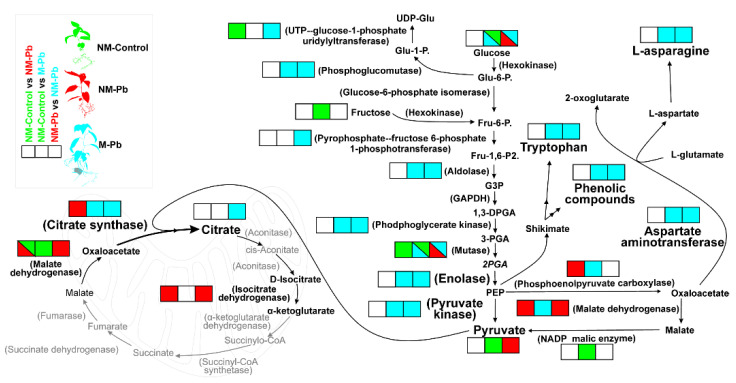
Primary metabolic alterations under Pb exposure. The color code (green: increased in NM-Control roots; red: increased in M-Pb roots; blue: increased in M-Pb roots; white: nonsignificant change or not detected) is used to represent changes in the abundances of the identified enzymes (involved mainly in glycolysis and TCA) and selected metabolites according to three independent T tests: NM-Control vs. NM-Pb (1), NM-Control vs. M-Pb (2) and NM-Pb vs. M-Pb (3; square boxes from left to right). The most important changes are marked with a larger font.

Most importantly, in M-Pb roots, we observed the increased abundances of glycolytic and TCA cycle enzymes (Figure 6), although proteomic signals of mitochondrial electron transport chain activation were not detected (Table S1). The source of the glycolytic intermediates also remained unknown. The source may be carbohydrates present in the growth medium preferentially taken up by M-Pb plants [61] rather than C-compounds provided by leaves, as suggested by the symptoms of reduced photosynthesis: lower levels of Mg, leaf pigments and N observed in M-Pb leaves (Figure S2; [62]). The main mobile carbohydrate transferred from leaves to sink organs, sucrose [63], was not increased in M-Pb poplar roots compared to that in all noninoculated poplars (data not shown), which was similar to that observed for the levels of glucose and fructose (Figure 6). Nevertheless, we detected clear OMIC signals of the redirection of metabolome flux into the glycolysis-mediated biosynthesis of phenolic compounds, amino acids and citrate (Figure 6).

Both phenolic compounds (known antioxidants; [64]) and amino acids increase the overall fitness of M-Pb poplars [65,66] and may bind Pb^2+^ ions [5,64], which would reduce the harmful concentration of free Pb ions in the plant cytosol [3,6,67].

However, the most important factor in the context of the Pb response seems to be the role of intensified citrate biosynthesis (Figure 6). Citrate is one of the most important low-molecular-weight organic acids known to play a vital role in chelating heavy metals in the rhizosphere, especially in ectomycorrhized plants [27,29,68]. Citrate secretion is usually linked with the increased activity of PM H^+^ ATPases [8,29,36,44,68], which are also upregulated in M-Pb plants (compared to both NM treatments). It was previously shown that ectomycorrhizal fungi under heavy metal stress may trigger the increased expression of various plant host genes, including H^+^ ATPases [29,36]. However, in the majority of reports, such increased low-molecular-weight organic acids and H^+^ ATPase abundances resulted in a decrease in the growth medium pH [10,29,36,69]. An increase in the acidity of the growth medium would increase Pb bioavailability, and indeed, increased HM uptake was frequently observed in ectomycorrhizal plants [8,19,29,36,70]. Surprisingly, in poplars with low levels of colonization, we observed a significantly increased agar pH. This effect was probably responsible for the lower Pb levels detected in M-Pb roots (above pH ~5.5, Pb bioavailability rapidly decreases; [71]), but this raises the question of the origin of the decrease in pH. The decreased acidity of the agar-grown medium could theoretically be caused by polyamines. Polyamines, which have been reported to be released by *P. involutus* hyphae in large amounts [72], are known regulators of plant growth. These basic molecules promote plant root growth regardless of ectomycorrhizal root colonization [38,73], exactly as observed in our study.

H^+^ ATPase activity generates a proton gradient throughout the plasma membrane [19,44]. The resulting ‘apoplastic protons’ may be theoretically used for H^+^/Pi symport [74]. Such a hypothesis would explain increased P level in M leaves. Protons could also be ‘consumed’ by the H^+^/citrate cotransport systems (e.g., the H^+^-ATPase-coupled MATE co-transport system, suggested to be a H^+^/citrate antiport; [44,69]). The resulting proton influx along with the citrate outflow [44,75] would partly explain the increase in agar pH in Pb-exposed roots [69]. Such theoretical citrate efflux and further chelation of Pb in the rhizosphere [27,29,68] could also contribute to, as observed in our study, the decreased Pb recruitment in M-Pb roots compared to that in NM-Pb roots. 

There is, however, also a possibility that, due to an increase in Pb^2+^ absorption and the toxicity threshold in hyphae compared to that in plant cells [76], Pb could have been taken up by a vast external *P. involutus* mycelium (Figure S1). It could also be speculated that the pool of bioavailable Pb in the M-Pb treatment group was further decreased by other Pb-chelating compounds known to be excluded by ECM plants (and the *P. involutus* hyphae itself), such as soluble proteins, metallothionines, or phenolic compounds [8,25,76,77,78]. Moreover, the dark pigmentation of the growth medium observed in the M-Pb treatment group (Figure S1) may suggest the transformation of the secreted phenolics into melanins [18,77] which are also known Pb chelators [79]. All these mechanisms were probably responsible for the decreased Pb levels detected in M-Pb plants.

In our study, Pb accumulation was detected, as commonly reported, mainly in roots because Pb is blocked by the endodermis by Casparian strips and must follow the symplastic route before Pb is translocated to leaves via vascular flow [5,80,81,82]. This known and very strong translocation restriction phenomenon was not disrupted in inoculated poplar roots. However, some differences in Pb flux were probably present in the aboveground part of M-Pb [5,29,75] as we found more Pb in M leaves despite the lowered Pb level in M roots and stems (and overall lower Pb content in M-Pb plants compared with NM-Pb). Unfortunately, the transport of Pb from roots into the aboveground parts of plants is still poorly understood. It is known, however, that Pb may compete with Ca for binding to divalent ion transporters [12,28], and foliar Ca was slightly decreased in M-Pb plants compared with NM-Pb (Figure S1), which could suggest cation competition plays some role in regulating foliar Pb levels. Moreover, the important role that the apoplastic/symplastic barrier plays in transporting Pb into the root xylem and further root-to-shoot translocation involves various PM ATPases [75]. In roots, we detected the increased abundance of various PM- and V-type H^+^ ATPases; thus, there is a high probability that other ATPases were also upregulated in M-Pb leaves [75].

## 4. Conclusions

In minimally colonized M-Pb poplars, we generally detected less Pb (which was more dispersed in the increased mass volume of inoculated plants), which suggests that inoculation with ectomycorrhizal fungi is beneficial for HM-stressed plants despite the low root colonization ratio [29,34,35,36]. Nevertheless, the decreased Pb uptake observed in bigger M-Pb plants coexisted with the intensive activation of molecular processes involved in Pb sequestration [83] or the redirection of the root metabolic flux into an increase in the production of Pb chelates such as citrate [44]. In this context, in inoculated but minimally colonized roots, the plant cell molecular response was more intense than that in noninoculated plants (and the opposite of that in massively colonized plants, where the fungal biofilter resulted in reduced proteome responses in plant host cells exposed to Pb; [53]) but more efficient than that in noncolonized plants. Inoculation with ectomycorrhizal fungi increases the host plant’s heavy metal tolerance (mainly via activation of molecular pathways associated with reducing Pb bioavailability/toxicity) regardless of low root colonization ratio.

## 5. Materials and Methods

### 5.1. Poplar Trees and Fungal Cultures

In our experiment, we compared three variants: nonmycorrhized (NM) plants grown in control conditions (NM-Control), nonmycorrhized plants exposed to Pb (NM-Pb) and inoculated poplars exposed to Pb (M-Pb). 

*Populus × canescens* microcuttings were cultivated in vitro in agar (0.8%) containing full-strength Murashige and Skoog medium (MS) supplemented with 1.5% sucrose covered with 3 mm of modified Melin–Norkrans medium (MNM) containing 1% glucose and 0.5% maltose. For the NM-Pb and M-Pb treatments, the agar medium was supplemented with 0.75 mM Pb(NO_3_)_2_. For the control treatment, nitrogen was compensated for with 0.75 mM NH_4_NO_3_. The final pH was adjusted to 5.5.

In the present study, we used *Paxillus involutus*, which increased in vitro poplar microcutting growth despite the low colonization ratio (Szuba, unpublished results). Fragments of barcoded (data not shown) *P. involutus* mycelium were placed near the freshly transferred poplar microcuttings growing in the two-layer agar medium.

Poplar cultures were grown in a growth chamber at 21 °C in 60% relative humidity with a 16 h/8 h day/night photoperiod using cool white fluorescent light (150 μmol m^−2^ s^−1^). Additionally, for the metabolomic study, pure cultures of *P. involutus* were cultivated in darkness in MNM medium on Petri dishes [18].

### 5.2. Harvesting and Biometrical, Biochemical and Root Colonization Analyses

Six weeks after inoculation, the leaves, stems and roots were weighed. Pooled samples of roots and agar medium as well as *P. involutus* hyphae (from the pure culture) were immediately frozen in liquid nitrogen and stored at –80 °C until analysis.

For the M-Pb treatment, the root colonization level was assessed. The root tip morphotype percentage was determined using ImageJ 1.48 v Software (Wayne Rasband, Bethesda, MD, USA) from high-resolution images captured during harvest. Categorization was performed on the basis of the anatomical structure of representative root tip morphotypes (selected during harvest and immediately analyzed under a microscope exactly according to [18]). Root tips were divided into three categories established during our previous study [18,61]: ‘nonmycorrhized’ root tips and two stages of root tip colonization, ‘changed’ root tips (root tips devoid of root hairs and covered with fungi—such root tips do not have the fully developed Hartig net) and ‘fully mycorrhized’ root tips (swollen, shortened root tips with a very well-developed mantle —such phenotypes have a well-developed mantle and Hartig net).

Pooled dried leaf (50 µg; we also analyzed the foliar P level; see Figure S1), stem and root (10 µg) samples were used to measure the Pb level in terms of both the concentration (μg DWg^−1^) and content (mg per plant). The Pb translocation factor was calculated according to formula: $\frac{\mathrm{Sum}\;\mathrm{of}\;\mathrm{Pb}\;\mathrm{contents}\;\mathrm{in}\;\mathrm{stem}\;\mathrm{and}\;\mathrm{leaves}\;\left(\mathrm{per}\;\mathrm{plant}\right)}{\mathrm{Root}\;\mathrm{Pb}\;\mathrm{content}\;\left(\mathrm{per}\;\mathrm{plant}\right)}.\;$ Mineral analyses were completed with six replicates using an inductively coupled plasma time-of-flight mass spectrometer (GBC Scientific Equipment, Hampshire, Braeside, Australia) as described in Szuba et al. [18].

Agar medium was agitated for 10 min at 4° C and then filtered through Miracloth (Millipore). The obtained supernatants were analyzed with an Elmetron CX-551 pH meter (n = 4).

### 5.3. Molecular Analyses

Concerning all molecular analyses, whole root systems were used for extractions. The proteome study was supplemented with metabolomic analysis to detect the final level of the compounds of interest. For molecular adjustments between NM-Control and M-control, see Szuba et al. [40].

#### 5.3.1. Proteome Analysis

Root proteins were isolated from 100 mg of pooled root sample according to the modified phenol extraction method described in Szuba et al. [61]. A minimum of five replicates were performed per treatment. The proteins were resuspended in buffer (4 M urea and 50 mM ammonium carbonate), and their concentrations were estimated using a 2-D Quant Kit according to the manufacturer’s procedure (GE Healthcare LS). Root protein extracts (10 µg of protein) were digested in solution using a standard method, and the obtained peptide solutions were used for each run (directly injected onto the LC column). Five biological repetitions per treatment were analyzed.

Peptide solutions were desalted and concentrated (using an RP C18 1 cm precolumn; Thermo Fisher Scientific, Waltham, MA, USA) prior to separation using a Dionex UltiMate 3000 RSLCnano System equipped with a 75 μm i.d. 25 cm RP C18 Acclaim PepMap column with a particle size of 2 μm and a pore size of 100 Å (Thermo Fisher Scientific). The Pierce LTQ ESI Positive Ion Calibration Solution (Thermo Fisher Scientific.) was used for system calibration. The following LC buffers were used: buffer A (0.1% (*v*/*v*) formic acid in Milli-Q water) and buffer B (0.1% formic acid in 90% acetonitrile). The peptides were eluted from the column with a constant flow rate of 300 nL min^−1^ and a linear gradient of buffer B from 5% to 65% over 185 min. The eluted peptides were analyzed using the Q-Exactive Orbitrap mass spectrometer (Thermo Fisher Scientific) operated in the data-dependent MS/MS mode. The instrument was operated with the following settings. The resolution was set to 70,000 for MS scans and 17,500 for MS/MS scans to increase the acquisition rate. The mass spectra were acquired in the range from 300 to 2000 *m/z*. The MS AGC target was set to 1 × 10^6^ counts, whereas the MS/MS AGC target was set to 5 × 10^4^. Dynamic exclusion was set with a duration of 20 s. The isolation window was set to 2 *m/z*. Protein identification and label-free data normalization were performed using MaxQuant 1.5.3.30 (Max Planck, Munich, Germany) prior to quantitation. The mass spectra were compared against the SwissProt database using the Viridiplantae taxonomy filter. The data were then evaluated, and the statistics were calculated using Perseus software (version 1.4.1.3, Max Planck Institute of Biochemistry, Martinsried, Germany). The normalized data were filtered for reverse identifications (decoy analysis), contaminants, and proteins identified only by sites. The LFQ intensities were transformed to log values and filtered for blanks in samples. Then, missing values were replaced with values calculated from a normal distribution (imputation). The obtained matrix was then used for statistical calculation (see below).

The identified proteins were classified according to COG (Clusters of Orthologous Groups of proteins) classes using the EggNOG database (http://eggnogdb.embl.de/#/app/home (accessed on 20 August 2019)). Additionally, the subcellular localization was predicted using UniProt [www.UniProt.org (accessed on 10 October 2019)] and TargetP 1.1 [http://www.cbs.dtu.dk/services/TargetP/ (accessed on 10 October 2019)] databases. Raw proteomic data has been deposited in the PRIDE/ProteomeXchnge with the project accession number PXD020049.

#### 5.3.2. Metabolomics Analysis (GC MS/MS)

The root metabolites for GC MS/MS were isolated from 5 mg of root and *P. involutus* mycelium by methanol extraction and were further derivatized in a standard manner with N-methyl-N-(trimethylsilyl)trifluoroacetamide (MSTFA) exactly according to Swarcewicz et al. [84]. The extracts were analyzed using a Pegasus 4D GCxGC-TOFMS system (Leco) equipped with a DB-5 ms bonded-phase fused-silica capillary column (30 m length, 0.25 mm inner diameter, 0.25 μm film thickness) (J&W Scientific Co., Folsom, CA, USA). Three biological repetitions per treatment were analyzed.

The mixture components were separated on a GC column using the following temperature gradient: 2 min at 70 °C and then 10 °C/min to 300 °C with a hold for 10 min at 300 °C (36 min in total). As a carrier gas, helium was used at a flow rate of 1 mL/min. One microliter of each sample was injected in splitless mode. For sample introduction, a PTV injector was used starting at 40 °C for 0.1 min, and after that, the temperature was raised at 6 °C/s to 350 °C. The transfer line and ion source temperatures were maintained at 250 °C. EI ionization was performed with 70 eV energy. Mass spectra were recorded in the mass range of 50–850 *m/z*.

LECO ChromaTOF software was used for data acquisition, automatic peak detection, mass spectrum deconvolution, retention index calculation and NIST library searches. Retention indexes (RI) for each compound were calculated based on the alkane series mixture (C-10 to C-36) analysis. Metabolites were identified by library searches (NIST and Fiehn libraries); the analyte was considered identified when the quality threshold was passed, i.e., at a similarity index (SI) above 700 and a matching retention index ± 10. Artifacts (alkanes, column bleed, plasticizers, MSTFA and reagents) were identified analogously and then excluded from further analyses. The obtained data were normalized against the sum of the chromatographic peak areas (using the TIC approach), and the resulting tables were transferred into Perseus software (Max Planck). The ion intensities were transformed to log values and filtered for blanks in samples. The missing values in the Perseus data table were replaced (by constant value (0) imputation), and such prepared matrixes were used for the statistical calculations (see below). Metabolomic raw data have been deposited to the European Centre for Bioinformatics and Genomics (Poznan, Poland) Bioserver.

### 5.4. Statistical and Bioinformatics Analyses

Statistical analyses of the biometric features of poplars and Pb levels (as well as leaf pigments and foliar mineral composition; see Figure S1) were performed using JMP Pro 13.0.0 software (SAS Institute Inc.). Values were considered significant according to T tests or ANOVA/post hoc HSD tests (α = 0.05).

Statistical calculations for the molecular data (proteomic and metabolomic analyses) were performed in Perseus software. The log values (for information on data matrix preparation, see above) were analyzed using two-sample T tests and/or multisample ANOVA (α = 0.05; FDR = 0.05). Only the significantly differentially abundant compounds were subjected to a hierarchical analysis. For clustering analysis, data were normalized using the Z-score algorithm.

## Supplementary Materials

Supplementary Materials are available online at https://www.mdpi.com/article/10.3390/ijms22094300/s1.
