# Supplementary material for: Pb Stress and Ectomycorrhizas: Strong Protective Proteomic Responses in Poplar Roots Inoculated with Paxillus involutus Isolate and Characterized by Low Root Colonization Intensity
Source: Int J Mol Sci. 2021 Apr 21;22(9):4300. doi: 10.3390/ijms22094300 (PMC8122328; doi:10.3390/ijms22094300)
Supplement: Supplementary file 1 [file ijms-22-04300-s001.zip › supplementary files/Fig. S1 Representative images of poplars grown in jars_IV2021.pptx]

## Slide 1
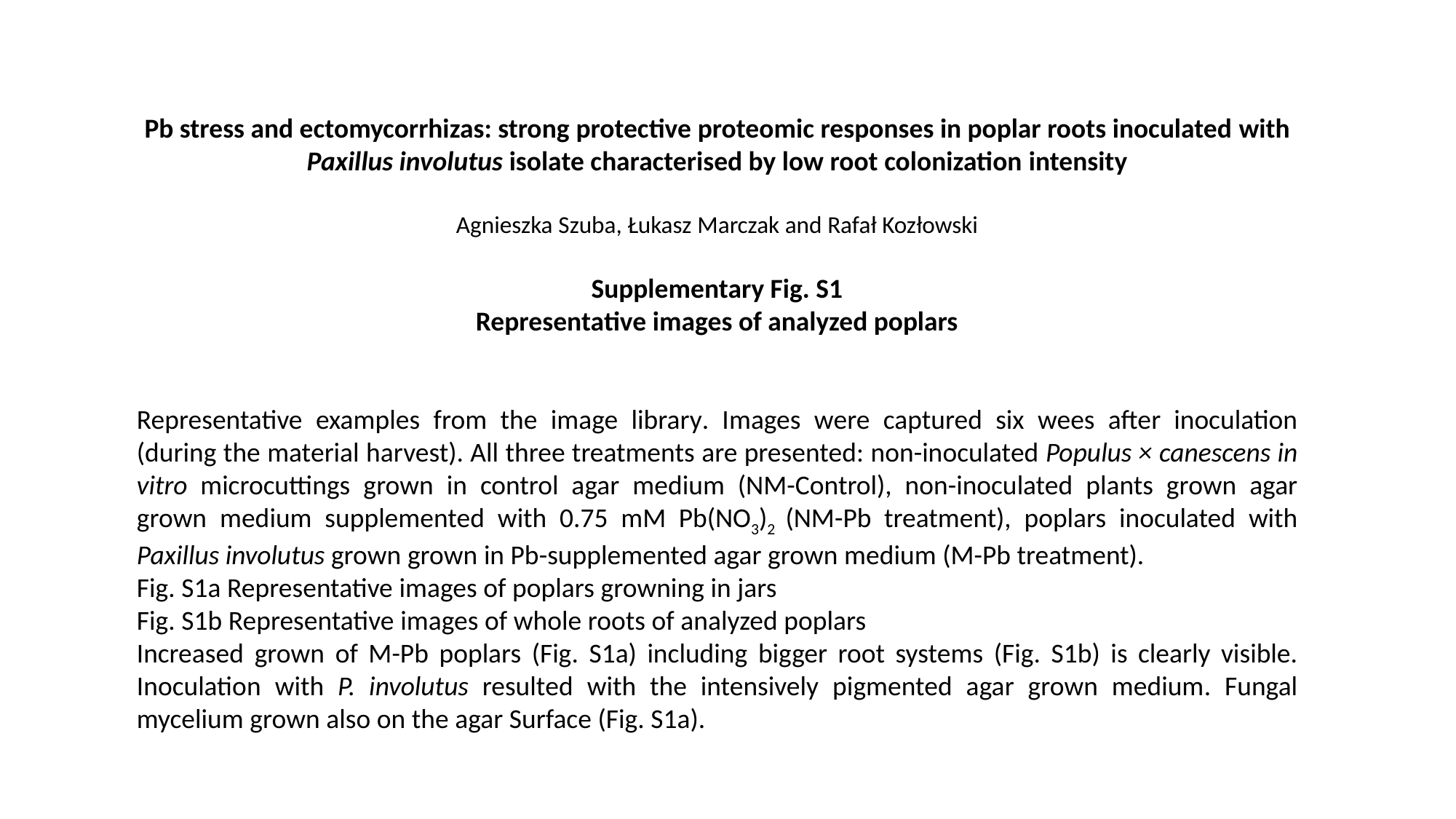

Pb stress and ectomycorrhizas: strong protective proteomic responses in poplar roots inoculated with Paxillus involutus isolate characterised by low root colonization intensity
Agnieszka Szuba, Łukasz Marczak and Rafał Kozłowski
Supplementary Fig. S1
Representative images of analyzed poplars
Representative examples from the image library. Images were captured six wees after inoculation (during the material harvest). All three treatments are presented: non-inoculated Populus × canescens in vitro microcuttings grown in control agar medium (NM-Control), non-inoculated plants grown agar grown medium supplemented with 0.75 mM Pb(NO3)2 (NM-Pb treatment), poplars inoculated with Paxillus involutus grown grown in Pb-supplemented agar grown medium (M-Pb treatment).
Fig. S1a Representative images of poplars growning in jars
Fig. S1b Representative images of whole roots of analyzed poplars
Increased grown of M-Pb poplars (Fig. S1a) including bigger root systems (Fig. S1b) is clearly visible. Inoculation with P. involutus resulted with the intensively pigmented agar grown medium. Fungal mycelium grown also on the agar Surface (Fig. S1a).

## Slide 2
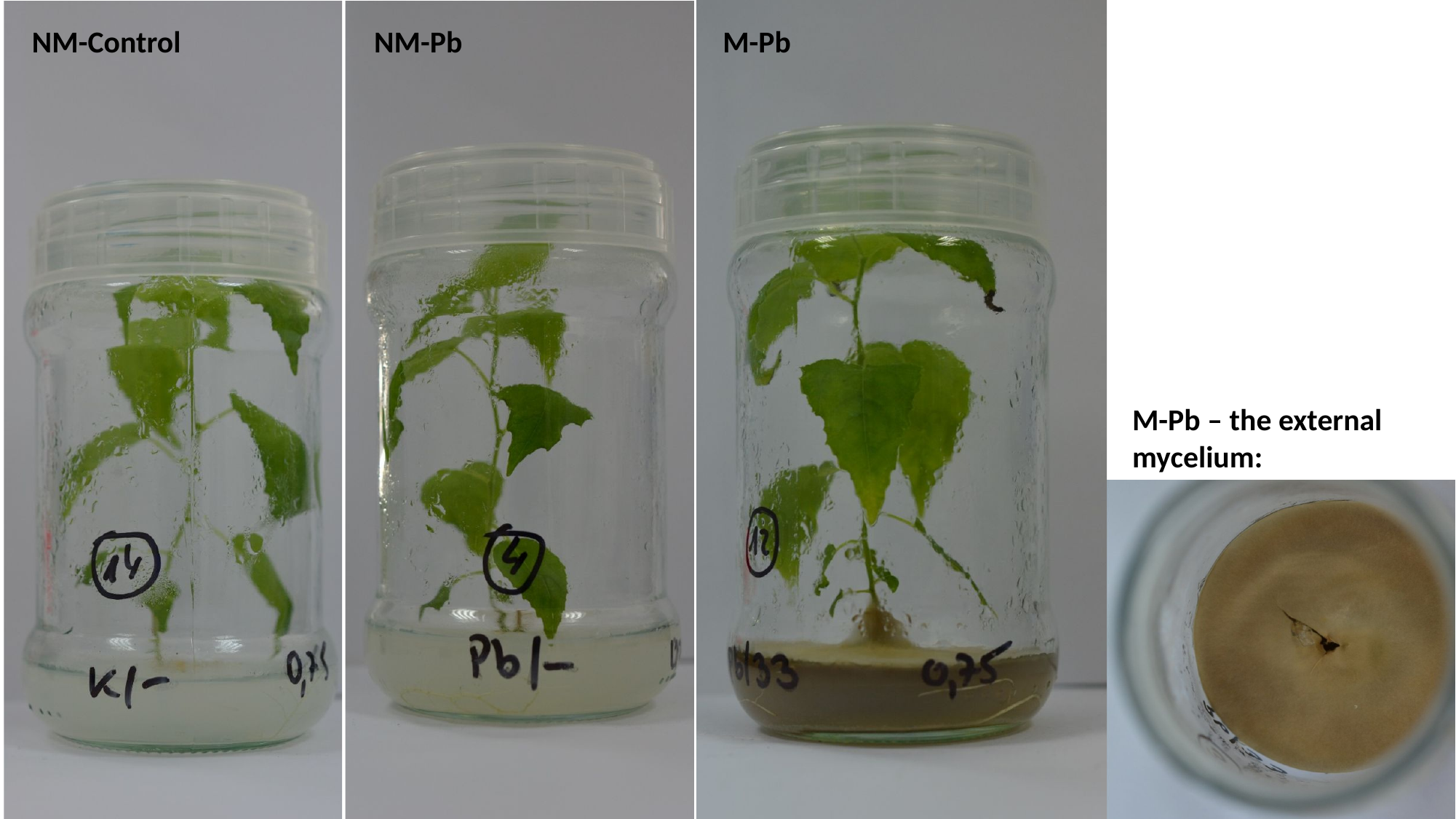

NM-Control
NM-Pb
M-Pb
M-Pb – the external mycelium:

## Slide 3
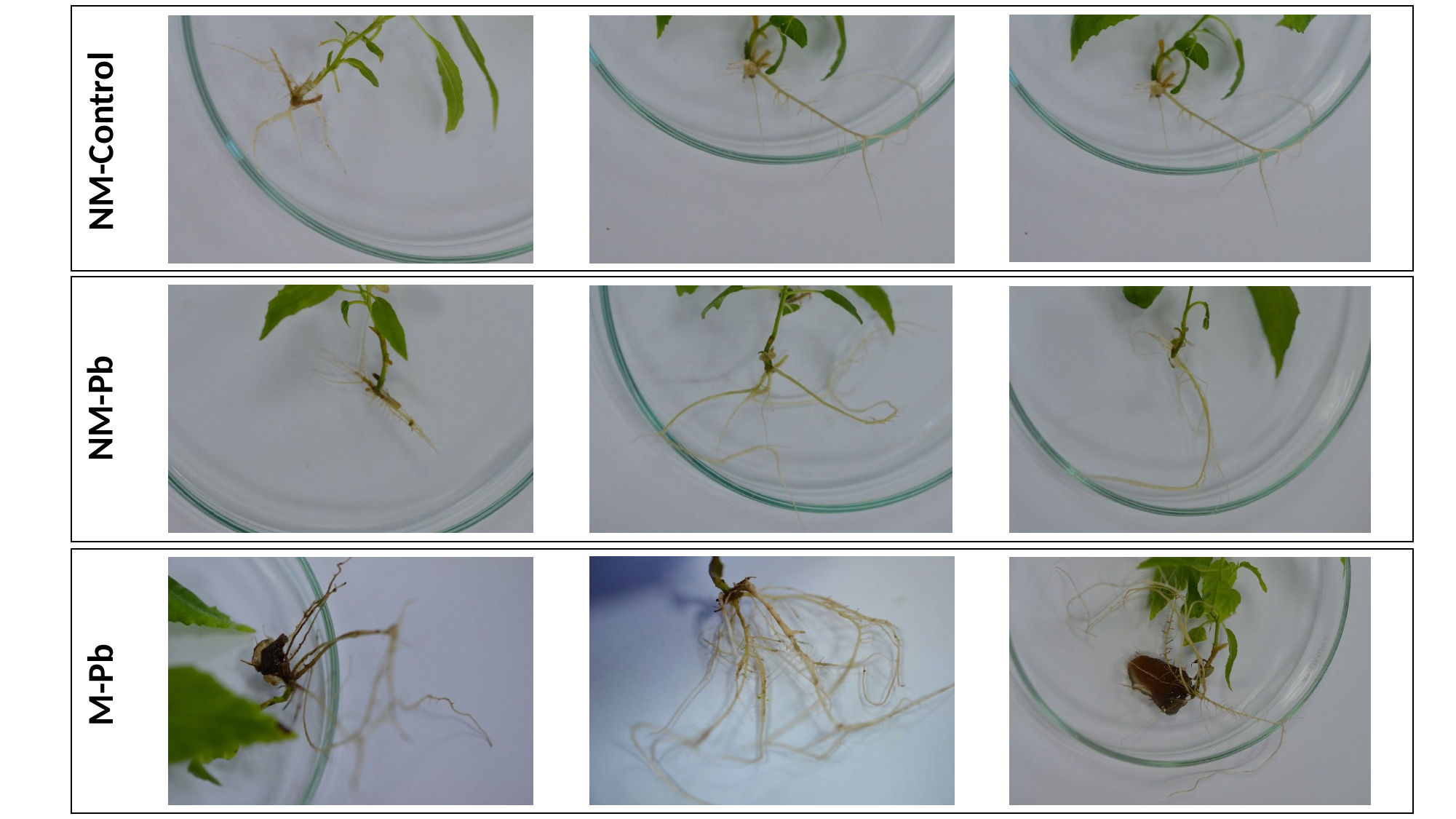

NM-Control
NM-Pb
M-Pb
