# Supplementary material for: Pb Stress and Ectomycorrhizas: Strong Protective Proteomic Responses in Poplar Roots Inoculated with Paxillus involutus Isolate and Characterized by Low Root Colonization Intensity
Source: Int J Mol Sci. 2021 Apr 21;22(9):4300. doi: 10.3390/ijms22094300 (PMC8122328; doi:10.3390/ijms22094300)
Supplement: Supplementary file 1 [file ijms-22-04300-s001.zip › supplementary files/Fig._S2_Foliar_biochemistry_IV2021.pptx]

## Slide 1
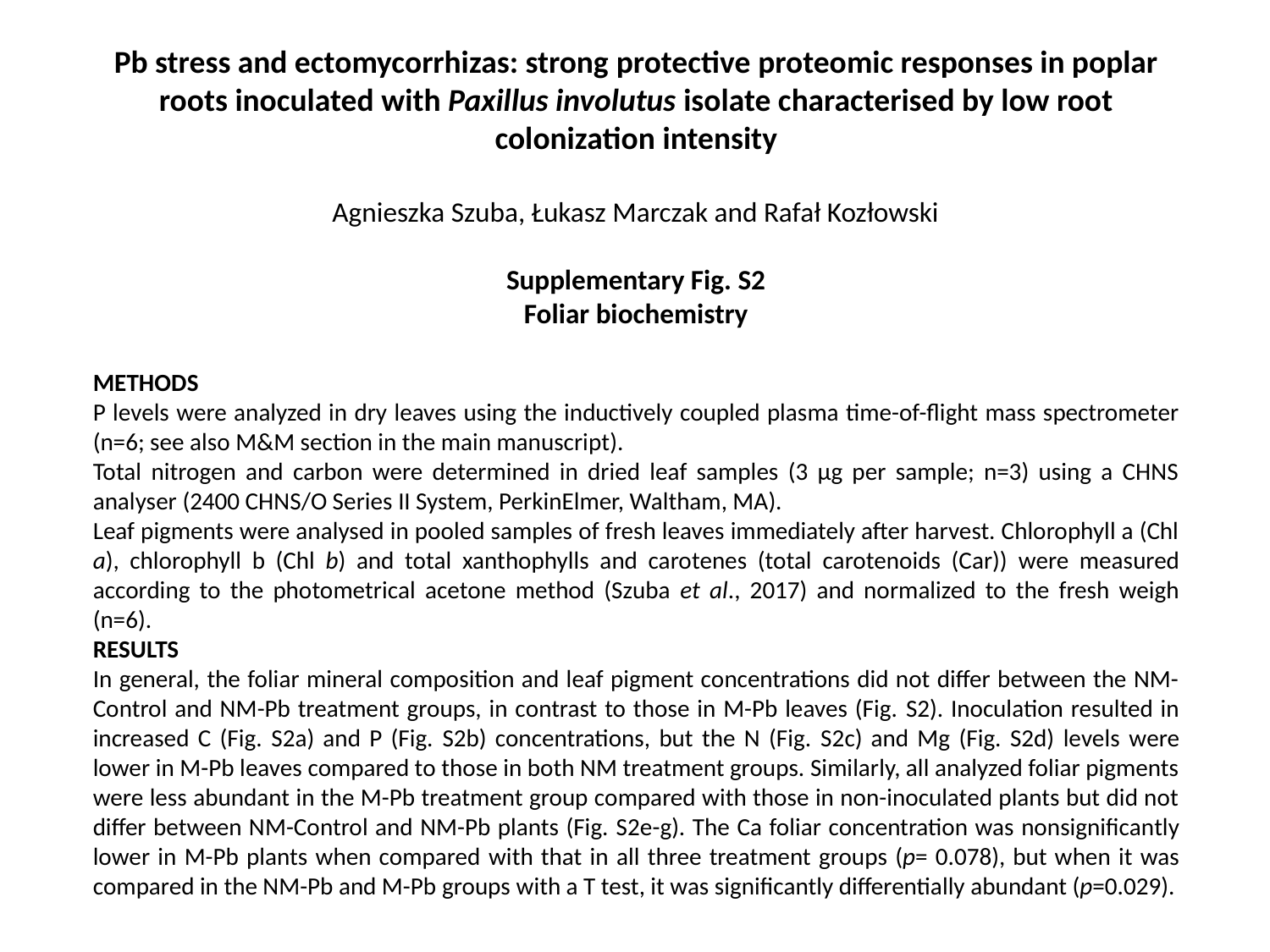

Pb stress and ectomycorrhizas: strong protective proteomic responses in poplar roots inoculated with Paxillus involutus isolate characterised by low root colonization intensity
Agnieszka Szuba, Łukasz Marczak and Rafał Kozłowski
Supplementary Fig. S2
Foliar biochemistry
METHODS
P levels were analyzed in dry leaves using the inductively coupled plasma time-of-flight mass spectrometer (n=6; see also M&M section in the main manuscript).
Total nitrogen and carbon were determined in dried leaf samples (3 µg per sample; n=3) using a CHNS analyser (2400 CHNS/O Series II System, PerkinElmer, Waltham, MA).
Leaf pigments were analysed in pooled samples of fresh leaves immediately after harvest. Chlorophyll a (Chl a), chlorophyll b (Chl b) and total xanthophylls and carotenes (total carotenoids (Car)) were measured according to the photometrical acetone method (Szuba et al., 2017) and normalized to the fresh weigh (n=6).
RESULTS
In general, the foliar mineral composition and leaf pigment concentrations did not differ between the NM-Control and NM-Pb treatment groups, in contrast to those in M-Pb leaves (Fig. S2). Inoculation resulted in increased C (Fig. S2a) and P (Fig. S2b) concentrations, but the N (Fig. S2c) and Mg (Fig. S2d) levels were lower in M-Pb leaves compared to those in both NM treatment groups. Similarly, all analyzed foliar pigments were less abundant in the M-Pb treatment group compared with those in non-inoculated plants but did not differ between NM-Control and NM-Pb plants (Fig. S2e-g). The Ca foliar concentration was nonsignificantly lower in M-Pb plants when compared with that in all three treatment groups (p= 0.078), but when it was compared in the NM-Pb and M-Pb groups with a T test, it was significantly differentially abundant (p=0.029).

## Slide 2
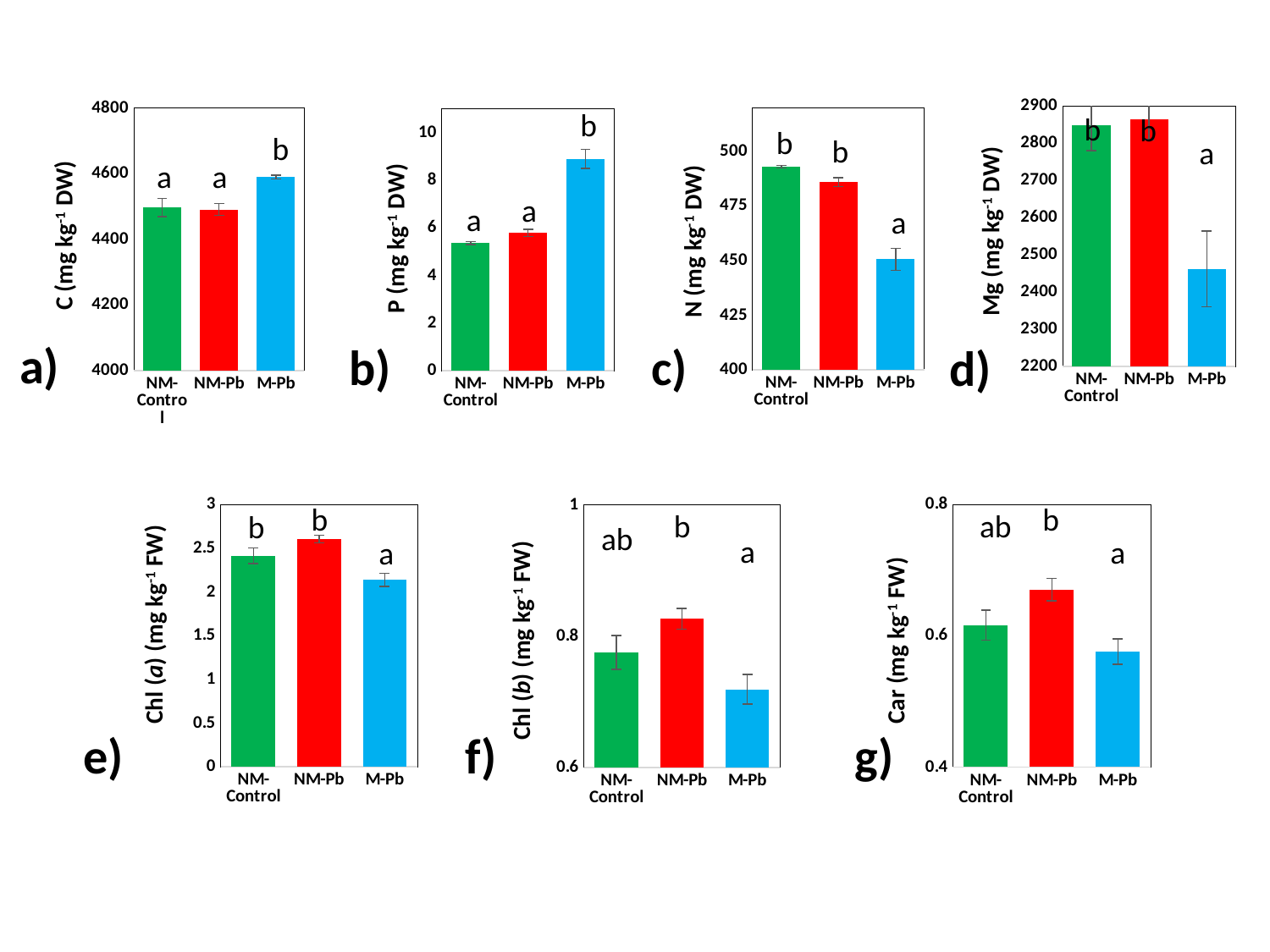

### Chart
| Category | Foliar Ndw |
|---|---|
| NM-Control | 493.0 |
| NM-Pb | 486.00000000000006 |
| M-Pb | 450.66667 |
### Chart
| Category | Foliar mg dw |
|---|---|
| NM-Control | 2847.8137 |
| NM-Pb | 2863.1513 |
| M-Pb | 2462.3768 |
### Chart
| Category | Foliar P dw |
|---|---|
| NM-Control | 5.352536199999999 |
| NM-Pb | 5.7873013 |
| M-Pb | 8.8920978 |
### Chart
| Category | Foliar C dw |
|---|---|
| NM-Control | 4497.0 |
| NM-Pb | 4491.0 |
| M-Pb | 4589.3333 |b
b
b
b
b
b
a
a
a
a
a
a
Mg (mg kg-1 DW)
C (mg kg-1 DW)
P (mg kg-1 DW)
N (mg kg-1 DW)
a)
b)
c)
d)
### Chart
| Category | Chl b |
|---|---|
| NM-Control | 0.6164415 |
| NM-Pb | 0.6704777 |
| M-Pb | 0.5761312 |
### Chart
| Category | Chl a |
|---|---|
| NM-Control | 2.4148062 |
| NM-Pb | 2.6074335 |
| M-Pb | 2.1381756 |
### Chart
| Category | Car |
|---|---|
| NM-Control | 0.7749738 |
| NM-Pb | 0.8265576 |
| M-Pb | 0.7190152 |b
b
ab
b
b
ab
a
a
a
Chl (a) (mg kg-1 FW)
Chl (b) (mg kg-1 FW)
Car (mg kg-1 FW)
e)
f)
g)
